# Supplementary material for: Neural population dynamics in songbird RA and HVC during learned motor-vocal behavior
Source: ArXiv. 2024 Jul 8:arXiv:2407.06244v1. Preprint. [Version 1] (PMC11261980)
Supplement: Supplement 1 [file NIHPP2407.06244v1-supplement-1.pdf]

Supporting information

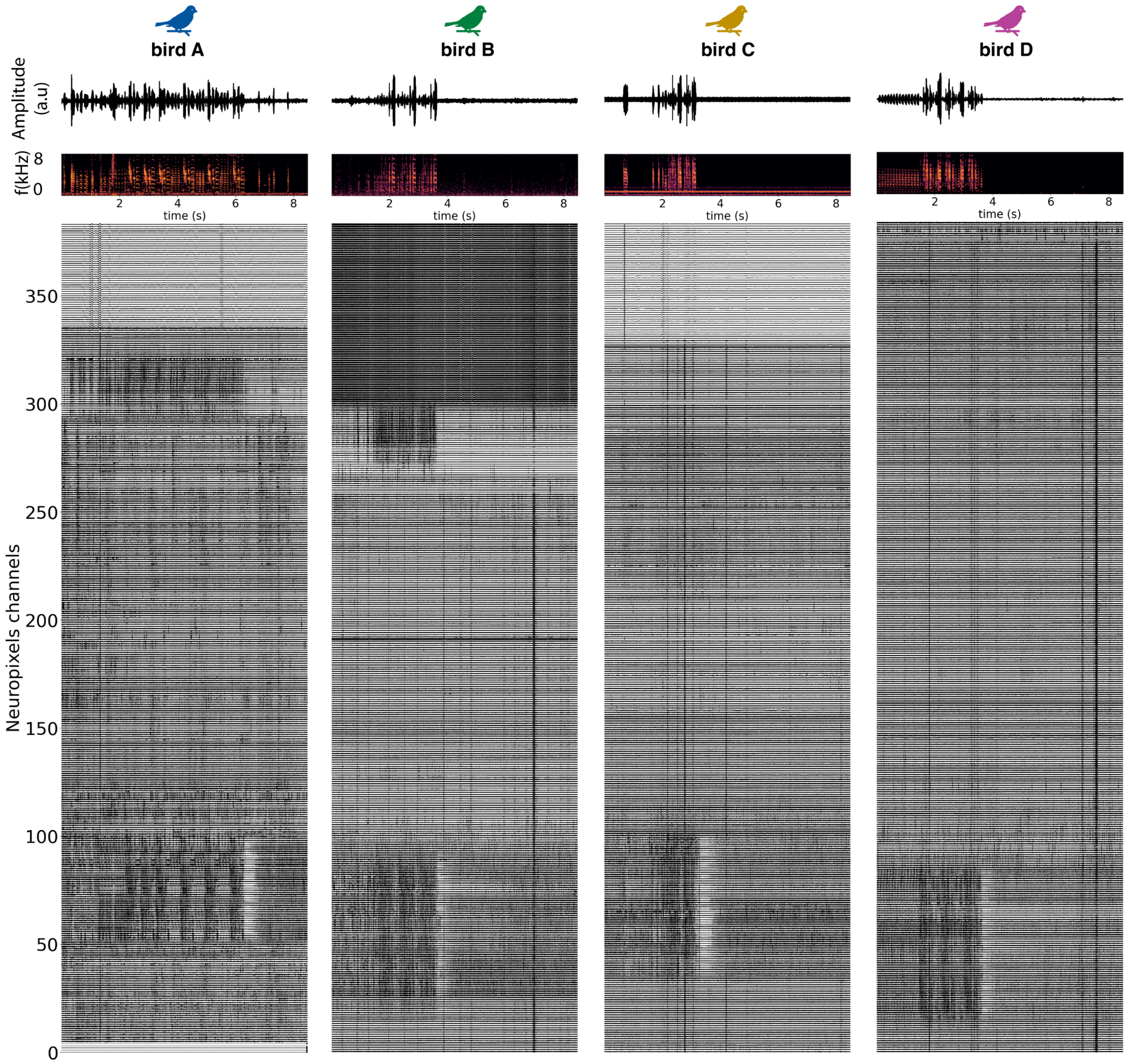

Figure S1. Examples of Neuropixels recordings time-locked to the production of song bouts in zebra finches. Each panel presents the audio waveform and corresponding spectrogram of a sample song bout for four different zebra finch exemplars. Below each, the corresponding neural traces recorded synchronously using a single Neuropixels probe are shown. RA population activity is identifiable in all four recordings, marked by a notable suppression of neural activity at the termination of the song bout. Birds A and B include HVC population activity. Bird C recordings solely capture RA activity. Bird D recordings contain only a few Neuropixels channels capturing HVC activity, insufficient for population-based analyses.

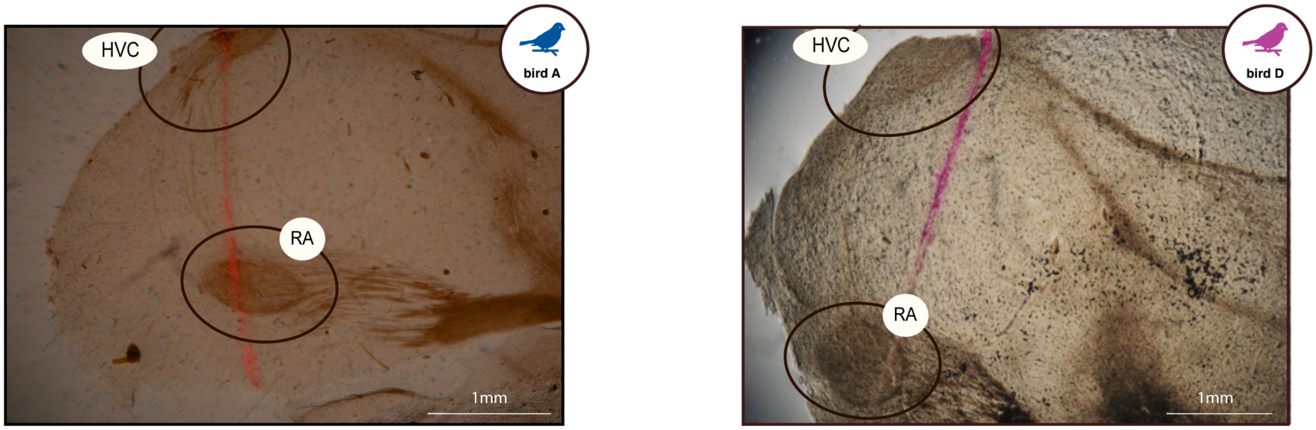

Figure S2. **Histological images of Neuropixels implants in zebra finches.** Bird A's histological image confirmed a successful implant across HVC and RA target regions. In contrast, the histological image for Bird D showed that while RA was successfully targeted, HVC was only tangentially targeted, resulting in only a few Neuropixels channels recording HVC activity.

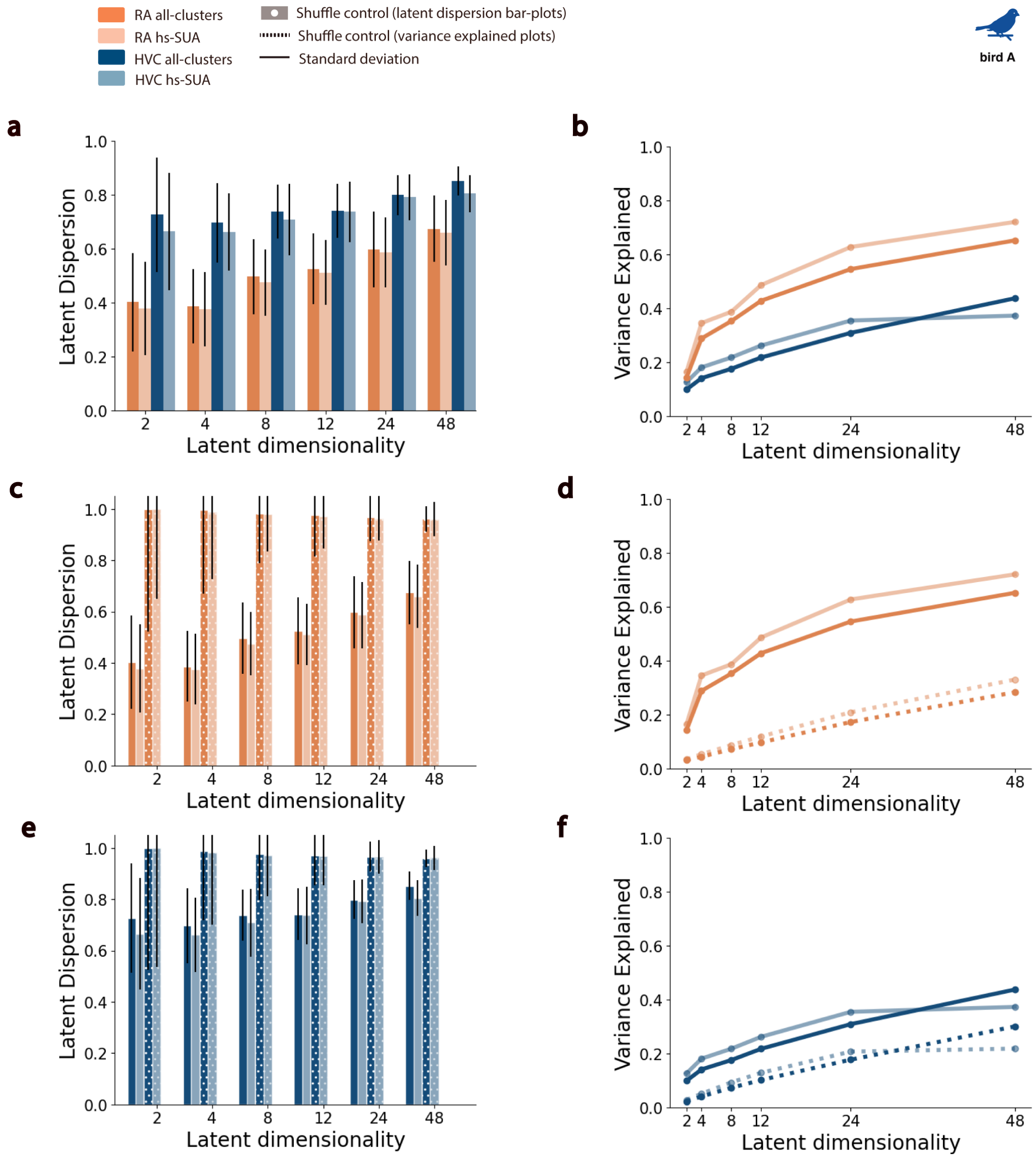

Figure S3. Bird A - Dimensionality-Dependent Analysis of Latent Neural Manifolds. a. Mean latent dispersion in GPFA-inferred neural manifolds as a function of latent dimensionality across *hs-SUA* and *all-clusters* HVC and RA neural populations. **b.** Neural variance explained by GPFA-inferred neural manifolds as a function of latent dimensionality across *hs-SUA* and *all-clusters* HVC and RA neural populations. **c.** Mean latent dispersion in GPFA-inferred neural manifolds as a function of latent dimensionality in RA neural populations and corresponding neural shuffle controls. **d.** Neural variance explained by GPFA-inferred neural manifolds as a function of latent dimensionality in RA neural populations and corresponding neural shuffle controls. **e.** Mean latent dispersion in GPFA-inferred neural manifolds as a function of latent dimensionality in HVC neural populations and corresponding neural shuffle controls. **f.** Neural variance explained by GPFA-inferred neural manifolds as a function of latent dimensionality in HVC neural populations and corresponding neural shuffle controls.

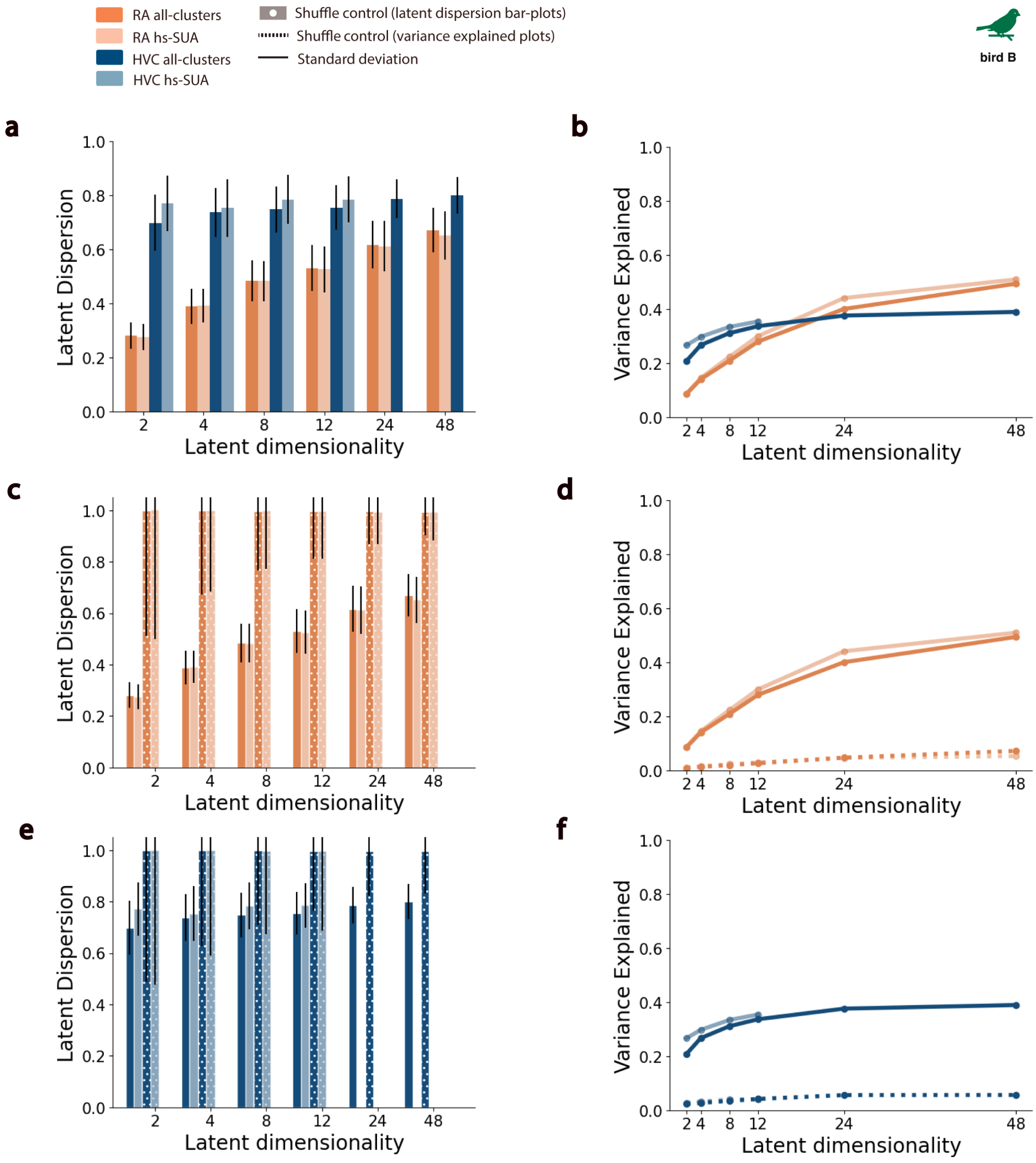

Figure S4. Bird B - Dimensionality-Dependent Analysis of Latent Neural Manifolds. a. Mean latent dispersion in GPFA-inferred neural manifolds as a function of latent dimensionality across *hs-SUA* and *all-clusters* HVC and RA neural populations. **b.** Neural variance explained by GPFA-inferred neural manifolds as a function of latent dimensionality across *hs-SUA* and *all-clusters* HVC and RA neural populations. **c.** Mean latent dispersion in GPFA-inferred neural manifolds as a function of latent dimensionality in RA neural populations and corresponding neural shuffle controls. **d.** Neural variance explained by GPFA-inferred neural manifolds as a function of latent dimensionality in RA neural populations and corresponding neural shuffle controls. **e.** Mean latent dispersion in GPFA-inferred neural manifolds as a function of latent dimensionality in HVC neural populations and corresponding neural shuffle controls. **f.** Neural variance explained by GPFA-inferred neural manifolds as a function of latent dimensionality in HVC neural populations and corresponding neural shuffle controls.

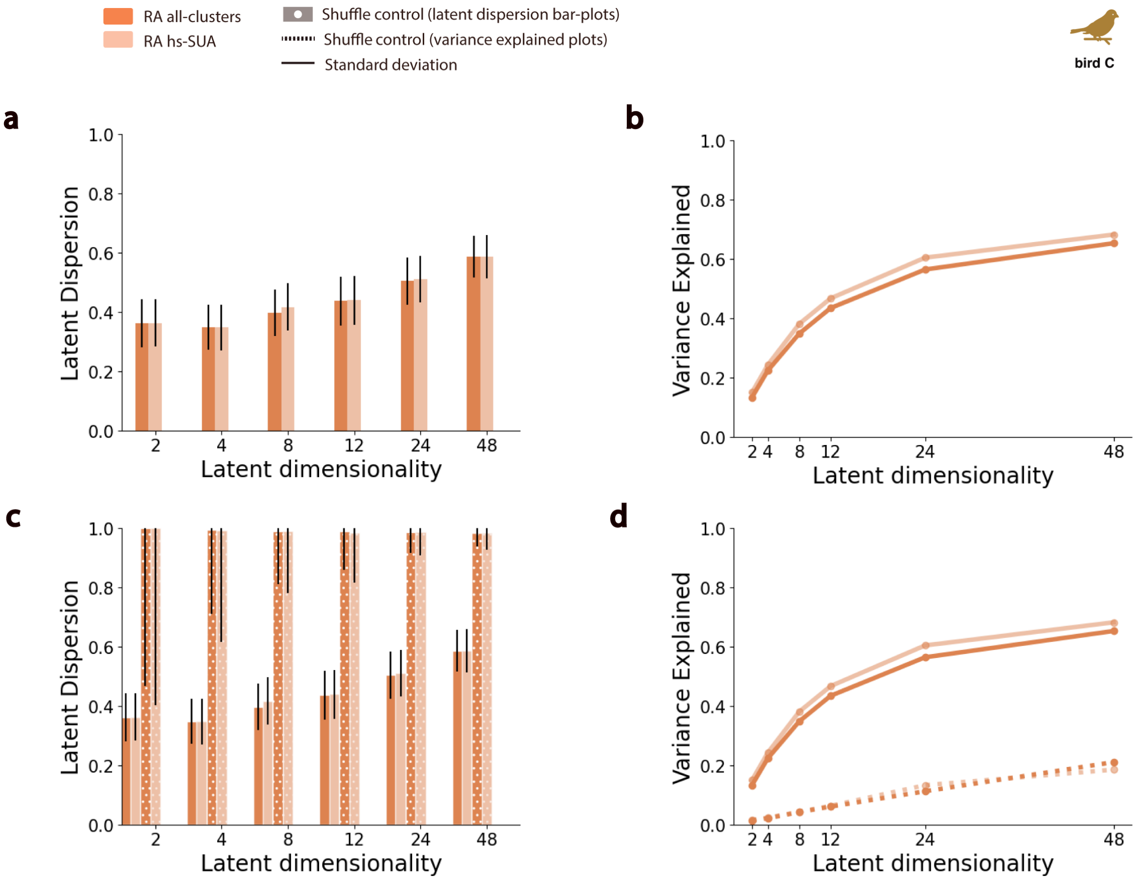

Figure S5. Bird C - Dimensionality-Dependent Analysis of Latent Neural Manifolds. a. Mean latent dispersion in GPFA-inferred neural manifolds as a function of latent dimensionality across *hs-SUA* and *all-clusters* RA neural populations. **b.** Neural variance explained by GPFA-inferred neural manifolds as a function of latent dimensionality across *hs-SUA* and *all-clusters* RA neural populations. **c.** Mean latent dispersion in GPFA-inferred neural manifolds as a function of latent dimensionality in RA neural populations and corresponding neural shuffle controls. **d.** Neural variance explained by GPFA-inferred neural manifolds as a function of latent dimensionality in RA neural populations and corresponding neural shuffle controls.

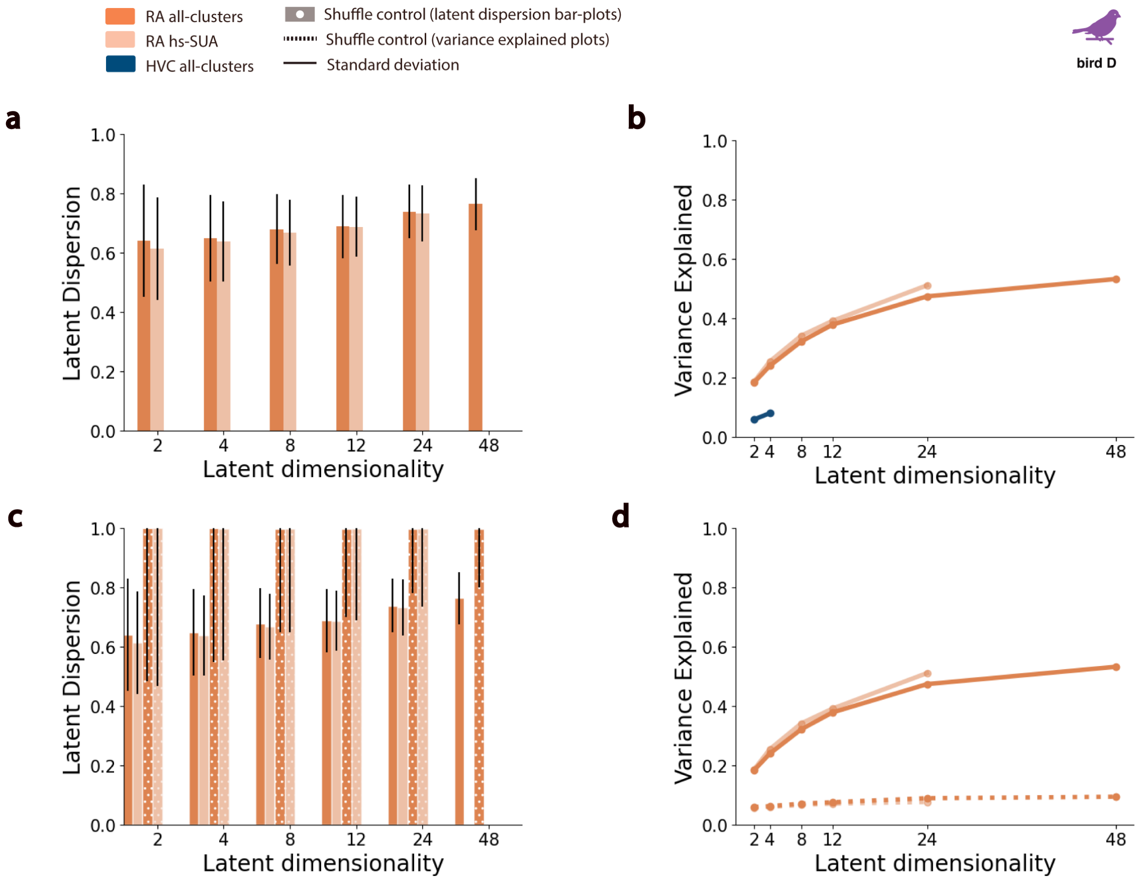

Figure S6. **Bird D - Dimensionality-Dependent Analysis of Latent Neural Manifolds. a.** Mean latent dispersion in GPFA-inferred neural manifolds as a function of latent dimensionality across *hs-SUA* and *all-clusters* RA neural populations. **b.** Neural variance explained by GPFA-inferred neural manifolds as a function of latent dimensionality across *hs-SUA* and *all-clusters* RA neural populations. **c.** Mean latent dispersion in GPFA-inferred neural manifolds as a function of latent dimensionality in RA neural populations and corresponding neural shuffle controls. **d.** Neural variance explained by GPFA-inferred neural manifolds as a function of latent dimensionality in RA neural populations and corresponding neural shuffle controls.

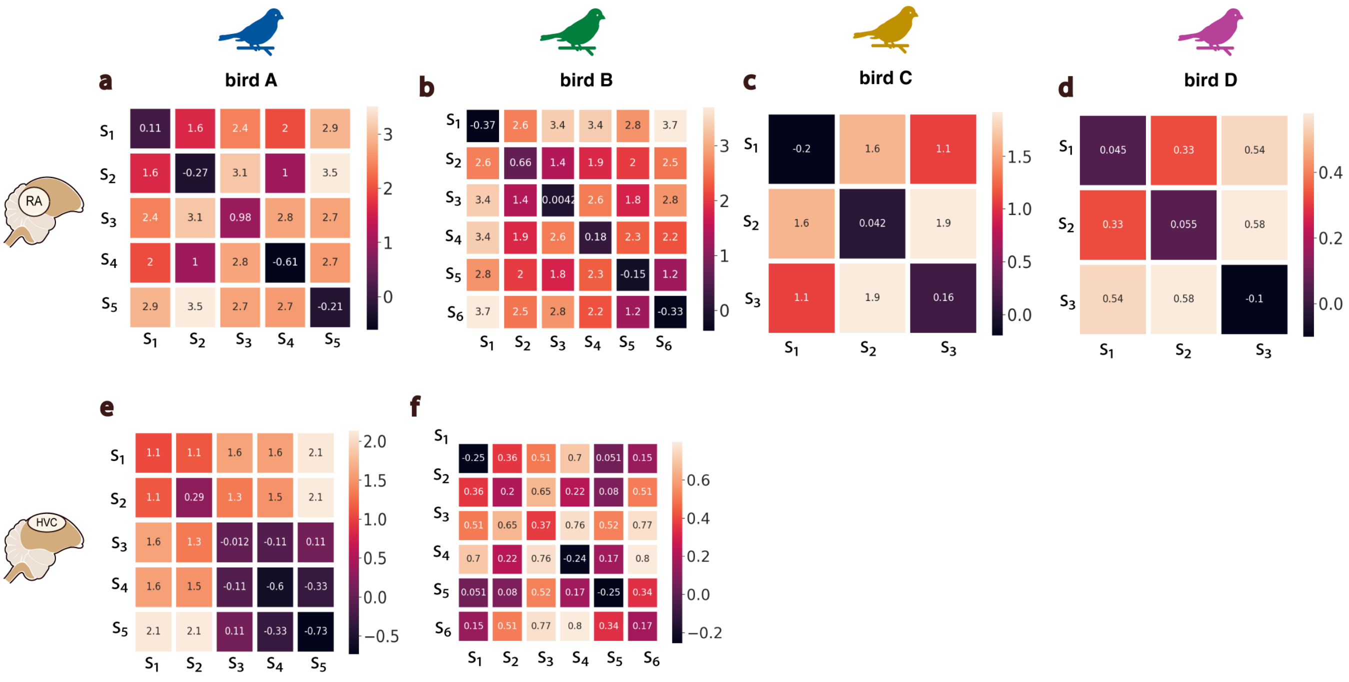

Figure S7. Syllable-based Latent Neural State Distance Confusion Matrices. Confusion matrices showing normalized, mean Euclidean distances in latent manifold across neural states corresponding to the production of different syllables in **a.** bird A - Ra **b.** bird B - RA **c.** bird C - RA **d.** bird D - RA **e.** bird A - HVC **f.** bird B - HVC.

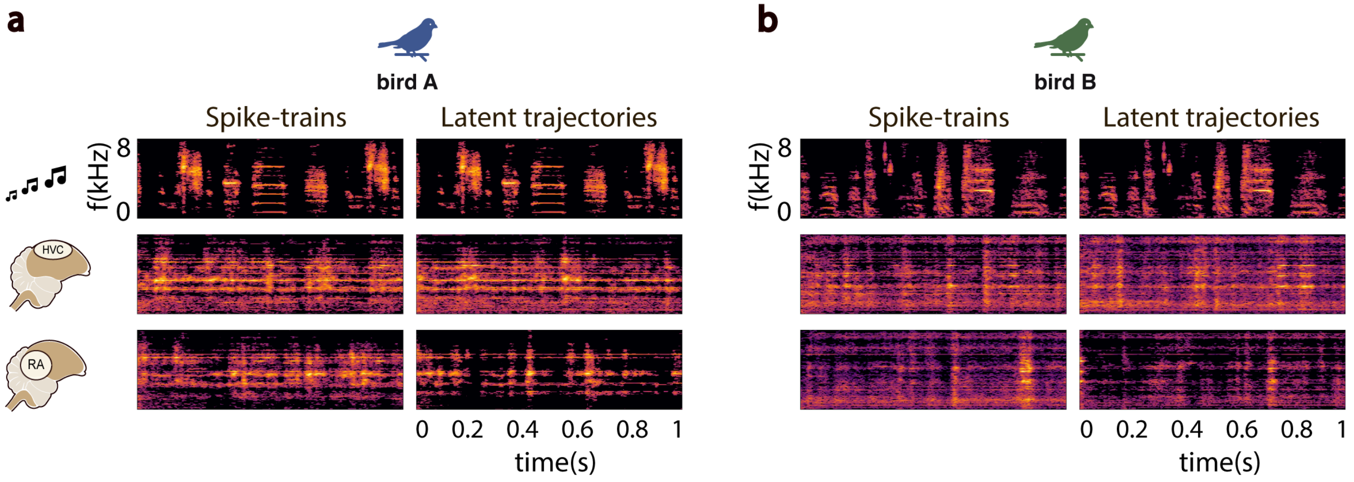

Figure S8. EnSongdec Controls - Synthesis of Audio Spectrograms from Shuffled Neural Activity. Synthesised spectrograms using HVC and RA shuffled spike-trains and GPFA-inferred latent states along the temporal dimension as inputs to EnSongdec in **a.** bird A and **b.** bird B.
